
JOURNAL INFORMATION
==============================
NLM Title Abbreviation: ScientificWorldJournal
Journal ID: ScientificWorldJournal
NLM Journal ID: 101131163
Journal ID: TSWJ

The Scientific World Journal

ISSN: 2356-6140
EISSN: 1537-744X
Publisher: Wiley

ARTICLE INFORMATION
==============================
PMCID: PMC5823115
PMID: 19882088
DOI: 10.1100/tsw.2009.140
Article ID: 542914
Article version: 1
Subjects: Clinical Images

Giant Spermatic Cord Lipoma

Edelstein Robert A. *
Merrimack Urology Associates, Chelmsford, Massachusetts, USA
*Robert A. Edelstein: rob.edelstein@comcast.netAcademic Editor: Daniel Batlle

Publication date: 2009
Electronic publication date: 2009 Nov 1
Volume: 9
First page: 1194
Last page: 1196
Received 2009 Sep 13; Revised 2009 Oct 19; Accepted 2009 Oct 21
Copyright: Copyright © 2009 Robert Edelstein.
Copyright year: 2009
License: This is an open access article distributed under the Creative Commons Attribution License, which permits unrestricted use, distribution, and reproduction in any medium, provided the original work is properly cited.
License URL: https://creativecommons.org/licenses/by/3.0/

Keywords: lipoma, spermatic cord

==============================
Clinical Image
*TheScientificWorldJOURNAL* (2009) 9, 1194–1196
ISSN 1537-744X; DOI 10.1100/tsw.2009.140

TheScientificWorldJOURNAL
www.thescientificworld.com

### Giant Spermatic Cord Lipoma

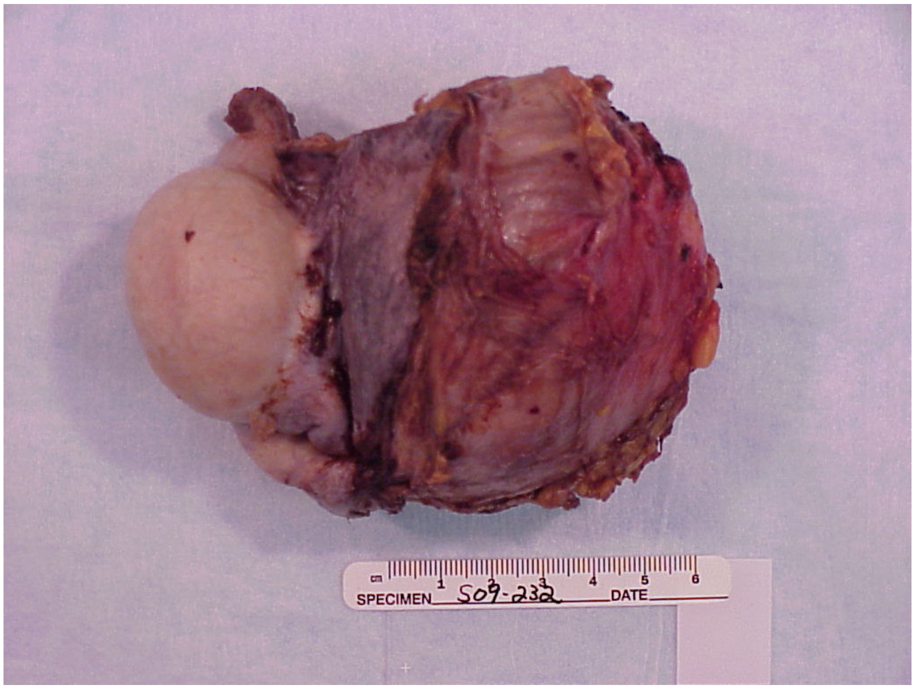

**FIGURE 1.** Gross appearance of spermatic cord. An associated hydrocele has been drained. Because of the patient's obesity, the large lipoma was well within the inguinal canal and defied preoperative palpation. The normal testis is shown at left, and the lipoma at right.

Robert A. Edelstein
*Merrimack Urology Associates, Chelmsford, Massachusetts*

**E-mail**: rob.edelstein@comcast.net

*Received September 13, 2009; Revised October 19, 2009; Accepted October 21, 2009; Published November 1, 2009*

**KEYWORDS**: lipoma, spermatic cord

A 48-year-old, obese male presented for the surgical repair of a large, ultrasound-documented hydrocele. He had undergone an uncomplicated ipsilateral hernia repair 8 years earlier and had gradually developed scrotal swelling. At the time of surgery, a hard, very large mass of the spermatic cord was identified (Fig. 1). The lesion could not be surgically separated from the cord and testis, necessitating en bloc orchiectomy. Pathologic analysis revealed the presence of a giant, benign, encapsulated lipoma of the

©2009 with author.
Published by TheScientificWorld; www.thescientificworld.com spermatic cord, without any evidence of recurrent hernia (Figs. 2 and 3). The etiology of such lesions is unclear, but may reflect development of local pluripotent cells into adipocyte lines.

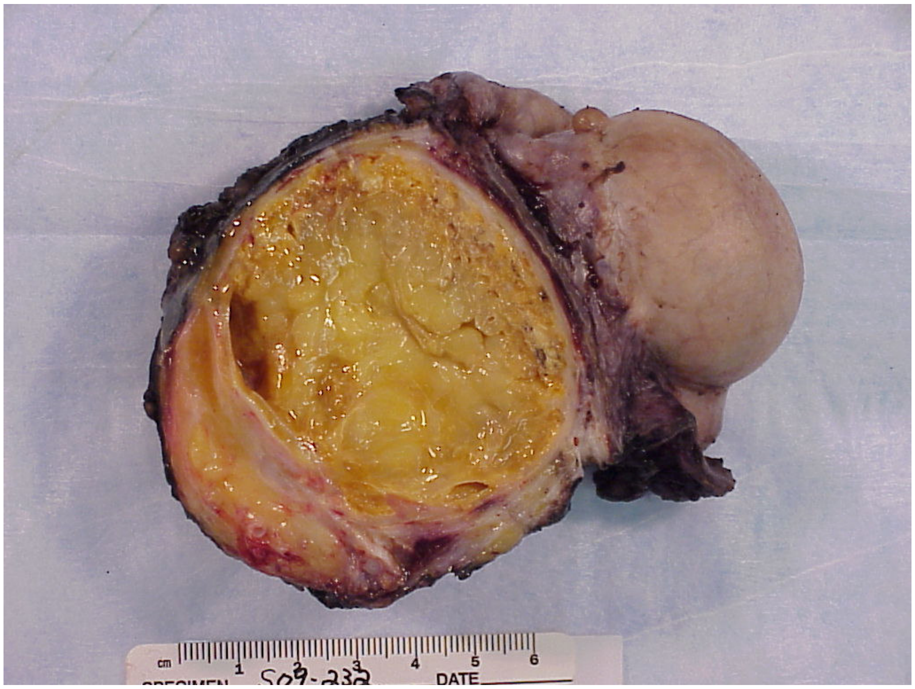

**FIGURE 2.** Cut section demonstrating relationship of lipoma to testis. The consistency of the lipoma (left side of picture, cut to expose interior) was quite firm. The normal testis is at right.

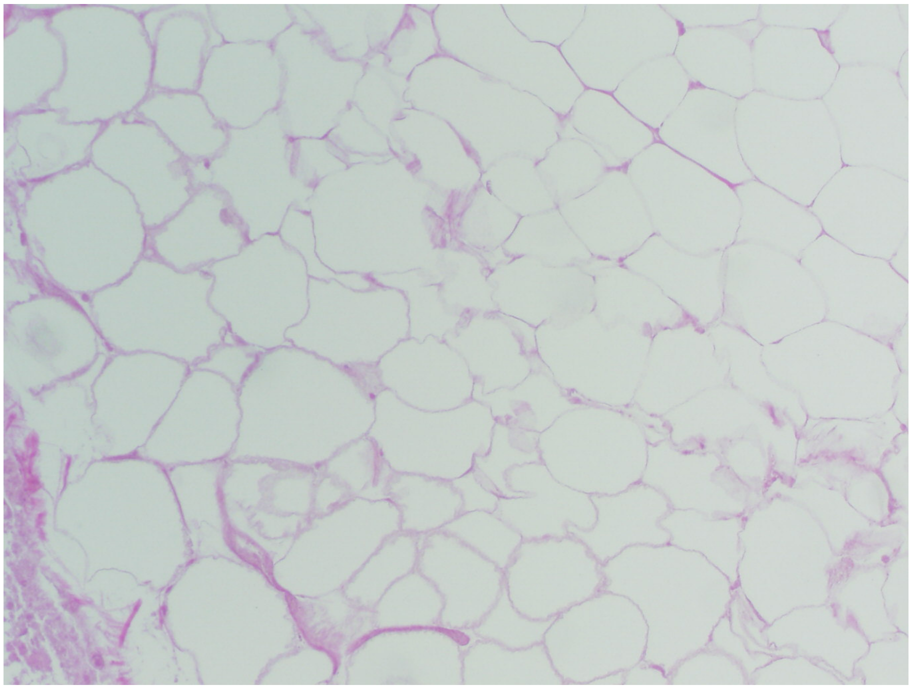

**FIGURE 3.** Histologic preparation demonstrating benign adipose tissue comprising the entirety of the lesion.

### REFERENCES

1. Lan, H., Loong, F., Yuen, W.K., and Patil, N.G. (2007) Management of herniated retroperitoneal adipose tissue during endoscopic extraperitoneal inguinal herniaplasty. *Surg. Endosc*. **21(9),** 1612–1616.
2. Read, R.C. and Schaefer, R.F. (2000) Lipoma of the spermatic cord, fatty herniation, liposarcoma. *Hernia* **4(3),** 149–154.

**This article should be cited as follows:**

Edelstein, R.A. (2009) Giant spermatic cord lipoma. *TheScientificWorldJOURNAL* **9**, 1194–1196. DOI 10.1100/tsw.2009.140.
